# The inappropriate use of time‐to‐independence biases estimates of activity patterns of free‐ranging mammals derived from camera traps

**DOI:** 10.1002/ece3.9408

**Published:** 2022-10-13

**Authors:** Christopher Peral, Marietjie Landman, Graham I. H. Kerley

**Affiliations:** ^1^ Centre for African Conservation Ecology Nelson Mandela University Gqeberha South Africa

**Keywords:** behavior, camera traps, daily activity, overlap, pseudoreplication, Serengeti

## Abstract

Measuring and comparing activity patterns provide key insights into the behavioral trade‐offs that result in animal activity and their extrinsic and intrinsic drivers. Camera traps are a recently emerged source of data for sampling animal activity used to estimate activity patterns. However, nearly 70% of studies using such data to estimate activity patterns apply a time‐to‐independence data filter to discard appreciable periods of sampling effort. This treatment of activity as a discrete event emerged from the use of camera trap data to estimate animal abundances, but does not reflect the continuous nature of behavior, and may bias resulting estimates of activity patterns. We used a large, freely available camera trap dataset to test the effects of time to independence on the estimated activity of eight medium‐ to large‐sized African mammals. We show that discarding data through the use of time‐to‐independence filters causes substantial losses in sample sizes and differences in the estimated activity of species. Activity patterns estimated for herbivore species were more affected by the application of time‐to‐independence data filters than carnivores, this extending to estimates of potential interactions (activity overlap) between herbivore species. We hypothesize that this pattern could reflect the typically more abundant, social, and patch‐specific foraging patterns of herbivores and suggest that this effect may bias estimates of predator–prey interactions. Activity estimates of rare species, with less data available, may be particularly vulnerable to loss of data through the application of time‐to‐independence data filters. We conclude that the application of time‐to‐independence data filters in camera trap‐based estimates of activity patterns is not valid and should not be used.

## Abstract 2

A rapidly growing body of literature is using data from camera traps to describe and compare activity patterns in animals, but most such studies arbitrarily apply an inappropriate data filter of removing activity records for a time after each sample used, the so‐called time to independence. Using data for eight African large mammals, we show that this approach is not conceptually valid and leads to biases in the estimated activity patterns and that tests for interactions (overlap) using such estimates may not be useful.

## 1 INTRODUCTION

The daily activity patterns of an animal reflect its phylogeny and the risks and rewards of activity or inactivity that determine fitness (Halle, 2000; Roll et al., 2006). Measuring and comparing activity patterns provides key insights into the behavioral trade‐offs that result in activity, such as food availability, mating opportunities, physiological processes, predation risk, and environmental constraints (Owen‐Smith, 1998; Tambling et al., 2015; Weyer et al., 2020; Zaman et al., 2022). These insights, together with the emerging availability of abundant activity data from camera traps, have led to renewed interest in describing the free‐ranging activity patterns of species and populations and comparing these between groups (e.g., predators and their prey, those at risk of predation vs. those not, and between time periods—Delisle et al., 2021; Diete et al.,  2017; O'Connell et al., 2011; Rowcliffe et al., 2014; Smith et al., 2020; Zaman et al., 2022).

**FIGURE 1:**
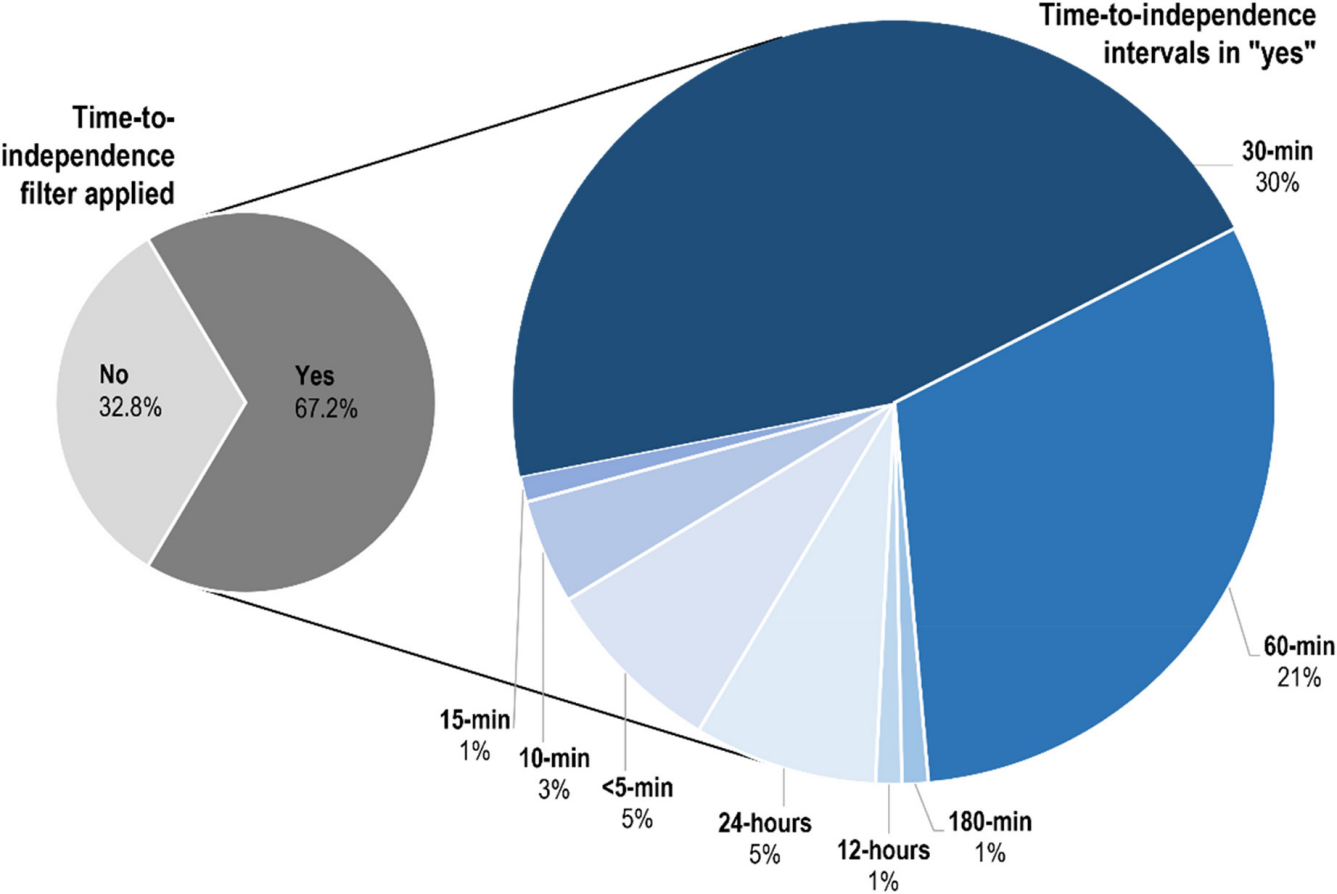
Distribution of the application of time‐to‐independence filters in 134 open‐access published studies (web of science, 1998–2021) that use camera traps to describe activity patterns in free‐ranging animals.

However, the trend in the literature is for such camera trap‐based estimates of activity patterns to approach activity as a discontinuous rather than a continuous state. This occurs by separating activity data (captured images) into discrete events by applying a time‐to‐independence filter and discarding all the images of a particular individual (or species) within this time‐to‐independence interval for each camera. Nearly 70% of the open‐access publications on free‐ranging animal activity patterns that we reviewed (Web of Science: 90 of 134 open access publications between 1998 and 2021) apply such a time‐to‐independence filter to camera trap data (Figure 1, Table S1). These filters are usually arbitrary (i.e., lacking a rationale), although avoiding pseudoreplication may be invoked (e.g. Zaman et al., 2022). They typically are 30 min duration, but may extend to 60 min and even 24 h. This can lead to discarding activity data for appreciable portions of the 24‐h cycle and will likely influence the ensuing estimates of activity patterns. This is analogous to the previously used approach of discarding autocorrelated location data in radio‐tracking studies, a practice that introduces biases in animal home range estimates (de Solla et al., 1999).

This time‐to‐independence filter approach contrasts strongly with traditional estimates of activity patterns that record and express activity as continuous and use all records of activity to quantify activity. This is epitomized by Aschoff (1954), who defined animal activity as “an animal is active when it moves parts of its body or moves itself.” Altmann (1974), in the classic study on measuring behavior, would define activity as a “state” (i.e., the animal is either active or inactive), not an “event,” and catered for measuring this through focal animal sampling that yields a continuous record of behavioral states (and the occurrence of events). There is an extensive body of literature that analyzes the activity patterns of animals, using, for example, data from direct observations (Davies & Skinner, 1986), records of animals breaking infrared light beams or altering conductance in an arena (Perrin, 1981; Smit & Langman, 1974), wheel running (Siepka & Takahashi, 2005), or implanted accelerometers that record movement (Weyer et al., 2020). All these studies use records of activity at the highest resolution (i.e., shortest interval between records) possible, and none of them discard activity records from their analyses.

How did this disjunction between established approaches for quantifying activity patterns and the camera trap time‐to‐independence approach come about? The use of time‐to‐independence filtering appears to stem from its use in determining animal abundances and densities, where multiple images of the same individual cause inflated estimates of abundance and density (Green et al., 2020; O'Connell et al., 2011; Wearn & Glover‐Kapfer, 2017). However, activity differs from the discrete nature of the occurrence of individuals or groups of animals. The absence of data to show that an animal is active during an observation period infers that it is inactive (the alternative state, following Altmann, 1974). Thus, the filtering and removal of activity data mean that the observer effectively decides that the animal is inactive in this period, discarding meaningful information on animal activity. This may lead to biases in our estimation of the activity patterns of animals and therefore also in our ability to detect changes in activity in response to conspecifics, predators, or competitors, food availability, or physiological constraints. Currently, there is no conceptual or empirical information on the influence of the time‐to‐independence approach on estimates of animal behavior (or more specifically, animal activity patterns) from camera traps.

Using a large, freely available camera trap dataset from the Serengeti National Park, Tanzania (Swanson et al., 2015), we explored whether the use of time‐to‐independence filters alters estimates of the activity patterns of eight African mammal species and interpretations of the interactions between these species. We chose these species to represent a suite of traits (Table 1), including mode of activity (diurnal, nocturnal, crepuscular), social structure (solitary, gregarious), and trophic guild (carnivores, herbivores). Our approach was to estimate the activity patterns of each species using different intervals of time to independence and then to compare these patterns within species and estimate interactions (overlap) between species across different times to independence. We hypothesize that the use of time‐to‐independence filters will result in an underestimation of activity within species and of overlap in activity between species. This would be particularly relevant during peak activity periods when records of activity (images) are frequent and occur close together in time, hence applying time‐to‐independence filters would discard the most data and bias estimates downward.

**TABLE 1:** The social structure and trophic guild, and the number of images extracted from the Snapshot Serengeti project dataset for each study species between June 2010 and May 2013, with the number of images remaining after the data were filtered according to six time‐to‐independence intervals (1–60 min).

Study species	Social structure	Trophic guild	Total no. images	Time interval					
				1‐min	5‐min	10‐min	15‐min	30‐min	60‐min
Wildebeest *Connochaetes taurinus*	Gregarious	Herbivore	100,660	100,179	20,395	14,594	12,159	9224	7319
Gazelle *Gazella thomsoni*	Gregarious	Herbivore	41,420	41,349	19,367	15,647	13,932	11,451	9563
Buffalo *Syncerus caffer*	Gregarious	Herbivore	13,672	13,444	5336	4504	4136	3792	3521
Eland *Tragelaphus oryx*	Gregarious	Herbivore	2689	2687	1015	989	966	883	801
Hyena *Crocuta crocuta*	Gregarious	Carnivore	5303	5303	3601	3461	3379	2906	2486
Serval *Leptailurus serval*	Solitary	Carnivore	458	458	395	390	388	378	364
Leopard *Panthera pardus*	Solitary	Carnivore	228	228	184	181	180	178	175
Caracal *Caracal caracal*	Solitary	Carnivore	79	79	63	63	63	62	61

## 2 METHODS

### 2.1 Dataset

To determine the influence of the interval of time to independence on mammal activity patterns, we used records of animal activity (images captured by motion‐triggered camera traps) collected by the long‐term Snapshot Serengeti project (Swanson et al., 2015, https://www.zooniverse.org/projects/zooniverse/snapshot‐serengeti). The project comprises 225 motion‐triggered camera traps placed in a 1125 km^2^ grid in the center of Serengeti National Park, Tanzania. Cameras are active throughout the 24‐h cycle, generally capturing bursts of images (three images per burst) within the first few seconds (1–10 s) of detected motion (Swanson et al., 2015). By May 2013, the project had produced ~1.2 million images, with the species identities classified by citizen scientists. We extracted data (comprising 164,509 images in total) on the eight mammal species collected between July 2010 and May 2013 for our study (Table 1).

We tested the influence of six time‐to‐independence intervals commonly used in the published literature: 1, 5, 10, 15, 30, and 60 min (Figure 1), with data from the longer intervals nested within the shorter intervals. Data for each interval were selected by sorting images by camera and species and removing images of the same species at a camera within the specified time interval. In all tests, we used the 1‐min time‐to‐independence interval (rather than 0 min) as the base case (or control) for comparison. This was necessary due to possible differences in the number and duration of detection bursts (used to improve species identifications—Forrester et al., 2016) between camera models (Swanson et al., 2015).

### 2.2 Data analysis

We used R 3.6.2 for all analyses (R Core Team, 2019) with a significance level of *α* = .05 for statistical tests. To assess the adequacy of our sample sizes, we plotted hourly accumulation curves of activity for each mammal species and time‐to‐independence interval. We visually assessed the shapes of the accumulation curves, expecting them to stabilize once the relationship between activity (i.e., records in the hour recorded as active) and the cumulative number of images reached an asymptote. Accumulation curves were fitted using the R‐library *vegan* (Oksanen et al., 2019). To determine where the accumulation curve reached an asymptote, we estimated breakpoints (two given the shape of the accumulation curve) with a segmented regression using the *segmented* library (Muggeo, 2010; Toms & Lesperance, 2003). Adequate sampling was achieved when the number of available images exceeded the number of images at the second breakpoint (i.e., at the asymptote).

To determine the daily activity patterns of each species for each interval of time to independence, we fitted non‐parametric kernel density functions (Meredith & Ridout, 2014; Ridout & Linkie, 2009). To delimit broad activity peaks (i.e., where records of activity are high), we visually assessed the shape of the activity density curves. Two broad shapes emerged (i.e., activity peak around midday and activity peaks around dawn and dusk), which roughly separated between the trophic guilds. Thus, for ease of comparison, we delimited hours of peak activity for the herbivores between 11:00 and 13:00 and for the carnivores between 05:00 and 07:00 and again between 20:00 and 22:00 (i.e., reflecting their crepuscular mode of activity). Activity peaks were then described as the number of images recorded in these peak periods of activity and expressed as a percentage of the total number of images.

The coefficient of overlap (∆), implemented in the *overlap* library, was used to estimate the degree of similarity in daily activity (Meredith & Ridout, 2014; Ridout & Linkie, 2009) within and between species and with time to independence. The coefficient of overlap ranges from 0 to 1, where 0 indicates no overlap in activity and 1 indicates complete overlap. Confidence intervals (95%) for coefficients of overlap were calculated using at least 1000 bootstraps. Model parameters were set according to the recommendations of Ridout and Linkie (2009) and Meredith and Ridout (2014) throughout. We compared pairwise overlap in activity patterns statistically with a Watson *U* ^2^ test (*circular* library; Agostinelli & Lund, 2017; Zar, 2010).

## 3 RESULTS

### 3.1 Adequacy of sample sizes

In total, we extracted 164,509 images from the Snapshot Serengeti Project dataset. Available sample sizes varied widely across species. These declined with the application of increasing intervals of time to independence (Table 1). In all cases, accumulation curves reached an asymptote, and the total number of available images exceeded the number of images at the asymptotes (i.e., the second breakpoint of the segmented regression; Table S2). This confirmed adequate sampling to describe the activity patterns of all study species for each interval of time to independence.

### 3.2 Within‐species effects

**FIGURE 2:**
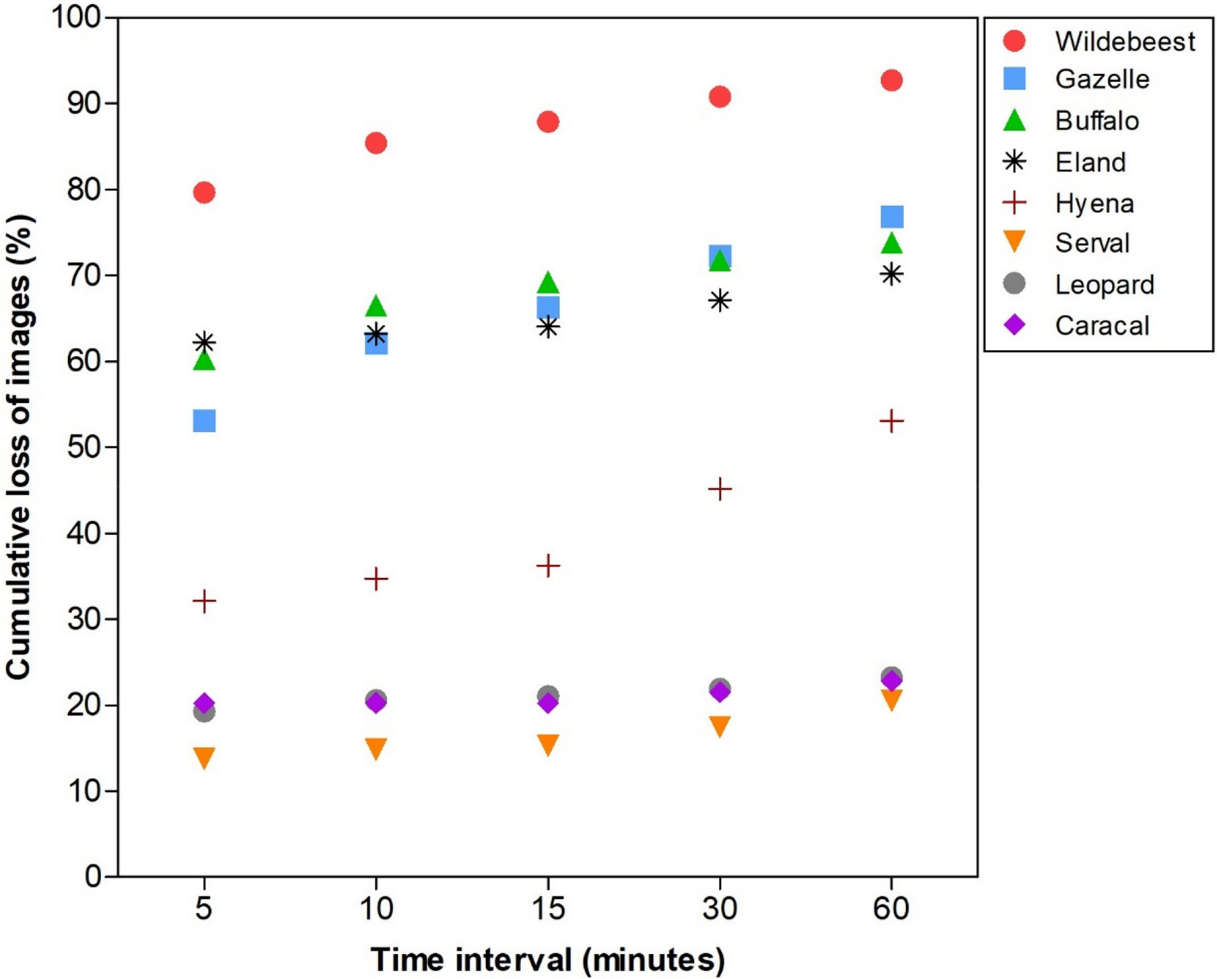
Cumulative loss of images (expressed as a percentage) for each study species with increasing interval of time to independence, using the 1‐min time‐to‐independence interval as the baseline.

Using time‐to‐independence filters predictably caused a loss of activity data across species (Table 1, Figure 2). However, the extent of the data loss varied between species: buffalo, gazelle, and wildebeest, species with large sample sizes, lost between 74% and 93% of their activity data when time to independence increased from 1 to 60 min. In contrast, species with smaller sample sizes, such as caracal, leopard, and serval, showed fewer data losses (between 21% and 23%) over the same time‐to‐independence intervals (Figure 2).

**FIGURE 3:**
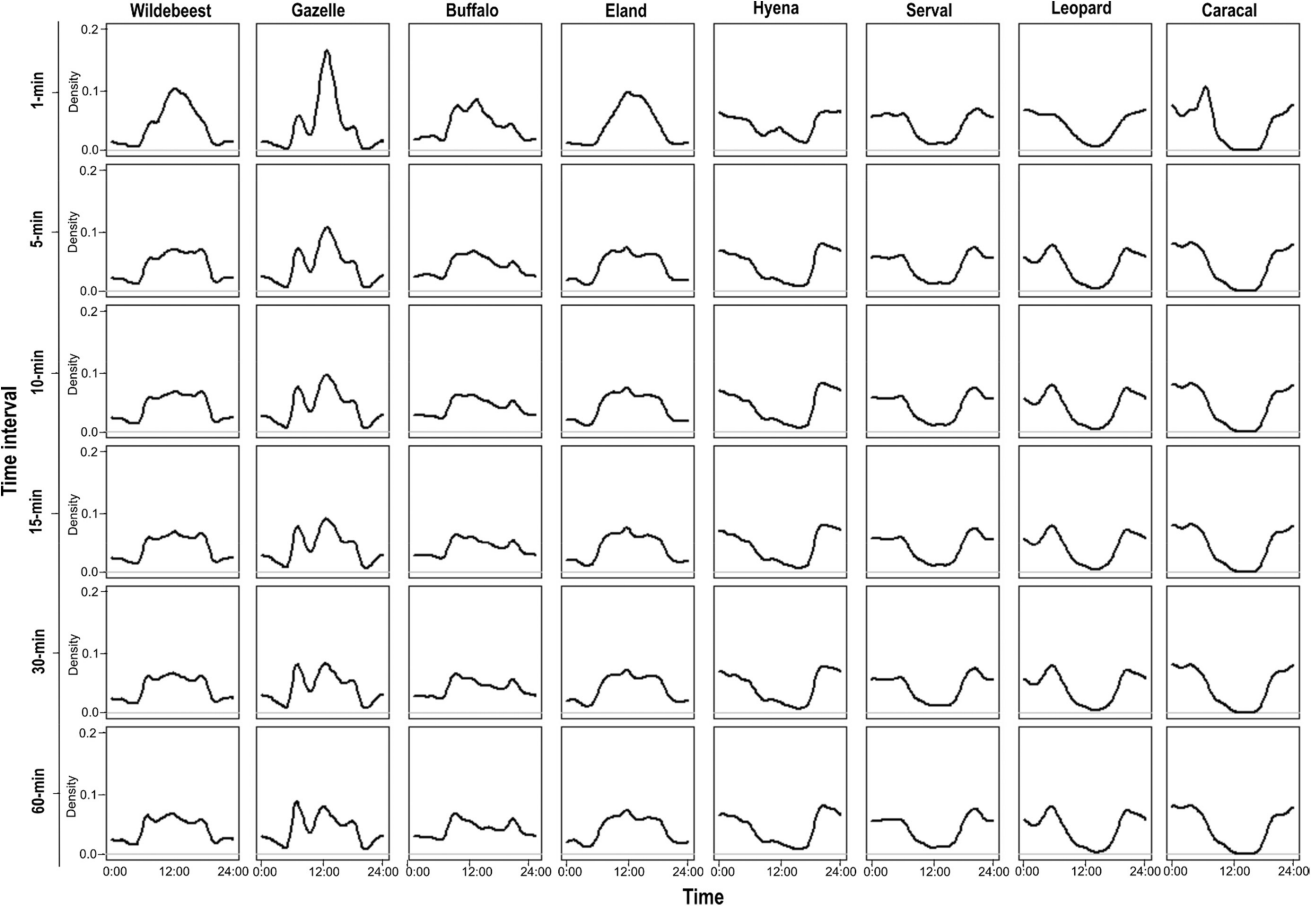
Daily activity density curves for the study species for each interval of time to independence.

As expected, discarding activity data caused changes to the estimated daily activity patterns (Figure 3). In particular, increasing the interval of time to independence dampened the broad activity peaks (i.e., where records of activity are high) of the herbivores, but accentuated these for the carnivores (Table 2). Wildebeest, gazelle, and buffalo, for example, showed a decline in their midday activity peak, this by as much as 44% in the case of gazelle when time to independence increased from 1 to 60 min. In contrast, hyena and leopard gained more defined activity peaks at dawn (increasing by 9% and 17%, respectively) and dusk (increasing by 18% and 16%, respectively) over the same increments of time‐to‐independence intervals. For the herbivores, the incremental dampening of peak activity with time to independence reduced the degree of overlap between the respective activity curves (generated for each time interval) and the control (i.e., the 1‐min interval) within each species (Table 3). That is, we detected a change in the overall activity pattern within each species with each time interval. In contrast, for the carnivores, the change in peak activity with time‐to‐independence filtering had little effect on overall activity patterns.

**TABLE 2:** Change proportion of images in the broad activity peaks estimated for each study species with time to independence, together with the percentage change in activity between the 1‐ and 60‐min intervals of time to independence.

Study species	Hours of peak activity	% Images in the activity peak						% ∆ Activity peak (1‐min:60‐min)
		1‐min	5‐min	10‐min	15‐min	30‐min	60‐min	
Wildebeest *Connochaetes taurinus*	11:00–13:00	19.1	13.0	12.6	12.7	12.6	12.7	−34
Gazelle *Gazella thomsoni*	11:00–13:00	26.2	18.1	16.6	15.7	14.9	14.6	−44
Buffalo *Syncerus caffer*	11:00–13:00	15.9	12.5	11.5	10.8	10.3	9.4	−41
Eland *Tragelaphus oryx*	11:00–13:00	19.1	14.0	14.3	14.5	13.7	14.4	−25
Hyena *Crocuta crocuta*	05:00–07:00	9.5	10.4	9.9	9.9	10.2	10.0	6
	20:00–22:00	12.9	15.2	15.7	15.6	15.3	15.8	22
Serval *Leptailurus serval*	05:00–07:00	13.3	13.4	13.3	13.1	13.0	12.4	−7
	20:00–22:00	15.1	14.9	15.1	15.2	15.3	15.7	4
Leopard *Panthera pardus*	05:00–07:00	14.9	17.4	17.7	17.8	18.0	17.7	19
	20:00–22:00	10.5	12.0	12.2	12.2	12.4	12.6	19
Caracal *Caracal caracal*	05:00–07:00	20.3	15.9	15.9	15.9	14.5	14.8	−27
	20:00–22:00	12.7	15.9	15.9	15.9	16.1	14.8	17

*Note*: Negative values indicate a dampening of the activity peak with increasing time to independence, and positive values indicate that the peaks are accentuated.

**TABLE 3:** Overlap coefficients and Watson's *U* ^2^ statistics for pairwise overlap comparisons of the activity curves of each study species given different intervals of time to independence against the 1‐min time‐to‐independence interval baseline.

Time interval	Statistic	Wildebeest	Buffalo	Gazelle	Eland	Hyena	Serval	Leopard	Caracal
5‐min	Overlap (95% CI)	0.85 (0.84–0.86)	0.92 (0.91–0.94)	0.85 (0.84–0.86)	0.85 (0.81–0.87)	0.90 (0.89–0.92)	0.93 (0.92–1.00)	0.89 (0.85–0.98)	0.83 (0.77–0.98)
	Watson's *U* ^2^	47.03	2.65	35.61	2.47	1.81	0.03	0.04	0.06
	*p*	<.001	<.001	<.001	<.001	<.001	>.10	>.10	>.10
10‐min	Overlap (95% CI)	0.84 (0.82–0.84)	0.90 (0.89–0.92)	0.81 (0.81–0.82)	0.85 (0.81–0.88)	0.90 (0.88–0.92)	0.93 (0.92–1.00)	0.89 (0.85–0.97)	0.83 (0.76–0.98)
	Watson's *U* ^2^	43.97	4.01	46.25	2.33	2.05	0.03	0.04	0.06
	*p*	<.001	<.001	<.001	<.001	<.001	>.10	>.10	>.10
15‐min	Overlap (95% CI)	0.83 (0.82–0.83)	0.89 (0.88–0.91)	0.80 (0.79–0.81)	0.86 (0.82–0.88)	0.90 (0.88–0.92)	0.93 (0.92–1.00)	0.89 (0.84–0.98)	0.83 (0.77–0.98)
	Watson's *U* ^2^	40.84	4.95	51.18	2.16	2.21	0.03	0.04	0.06
	*p*	<.001	<.001	<.001	<.001	<.001	>.10	>.10	>.10
30‐min	Overlap (95% CI)	0.82 (0.80–0.82)	0.88 (0.86–0.89)	0.77 (0.76–0.78)	0.84 (0.80–0.87)	0.90 (0.88–0.92)	0.93 (0.92–1.00)	0.89 (0.85–0.97)	0.83 (0.76–0.98)
	Watson's *U* ^2^	36.29	5.93	57.32	2.41	1.78	0.03	0.04	0.06
	*p*	<.001	<.001	<.001	<.001	<.001	>.10	>.10	>.10
60‐min	Overlap (95% CI)	0.81 (0.80–0.82)	0.87 (0.85–0.88)	0.75 (0.74–0.76)	0.85 (0.81–0.87)	0.90 (0.89–0.92)	0.93 (0.91–1.00)	0.89 (0.85–0.97)	0.83 (0.76–0.98)
	Watson's *U* ^2^	32.30	6.96	58.97	2.12	1.57	0.03	0.03	0.06
	*p*	<.001	<.001	<.001	<.001	<.001	>.10	>.10	>.10

### 3.3 Between‐species effects

To explore whether using time‐to‐independence filters alters interpretations of the potential interactions between species, we overlayed the activity patterns of wildebeest × gazelle, wildebeest × eland, and buffalo × eland for each interval of time to independence. These species combinations were selected to demonstrate the range of potential effects of time to independence on interpretations of species interactions, as expressed by overlap in activity patterns. We included no carnivores here because we observed no striking changes in activity between our study species with changing time to independence (Table 3).

**FIGURE 4:**
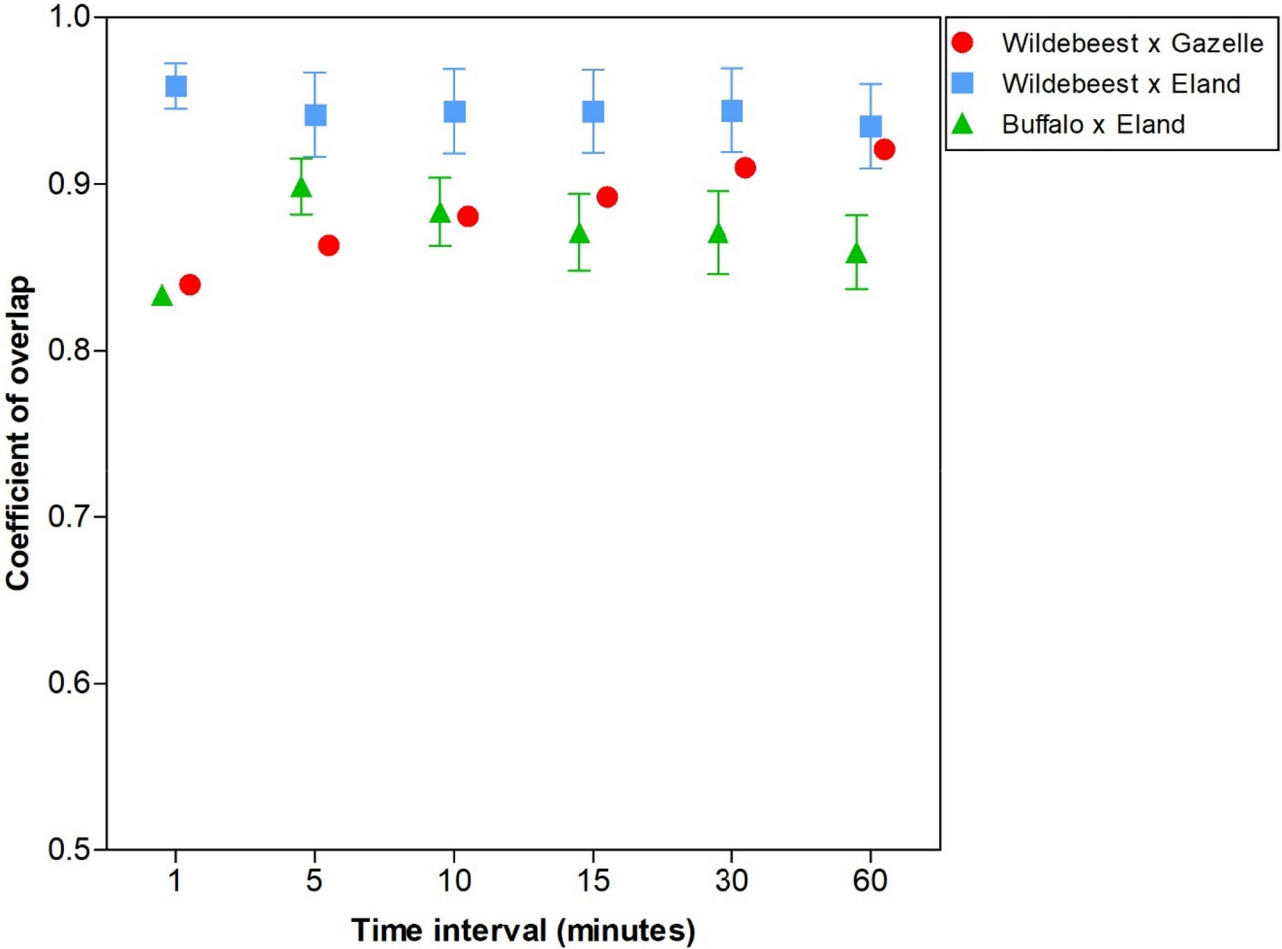
Effect of time‐to‐independence filters on overlap in activity between select study species.

For the herbivores, activity overlap varied between the different species combinations and with time to independence (Figure 4). Activity overlap between wildebeest × gazelle increased steadily with an increase in time to independence, while activity overlap between wildebeest × eland appeared to be more resilient to the effects of the time interval. In contrast, activity overlap between buffalo × eland responded initially, but later appeared to stabilize with an increase in time to independence.

## 4 DISCUSSION

Using a large, freely available camera trap dataset, our study explored the effects of time‐to‐independence data filters on the estimation of activity patterns in eight African large mammal species. We show that the application of these data filters alters our understanding of activity patterns of species and potential interactions between species and that these effects vary between species. The unusually large number of camera traps (225 traps) on which our study is based likely reduced the effect of discarding data in any time period (e.g., an animal's activity peak), as near‐contemporaneous activity data may have been collected for that species by another camera in the array. This suggests that studies using fewer camera traps (most such studies use fewer than 50) would be more vulnerable to the effects of discarding data through time‐to‐independence filters.

The application of time‐to‐independence filters causes the loss of activity data. In our study, herbivores lost more data than carnivores, and this was particularly striking during peak periods of activity (Figures 2 and 3, Tables 1 and 2). While the mechanism of this guild‐level effect is not immediately clear, this likely reflects a combination of the effect of sample size and the distribution of samples over each 24‐hr cycle, both of which varies with the species' life history traits, social structure, and patch use. In the case of sociality, for example, these herbivores are typically more social and occur together in larger groups than the studied carnivores (Estes, 2012). This means that captured images on a camera can accumulate relatively quickly within specific time intervals during peak periods of herbivore activity, leading to large sample sizes and hence a substantial cumulative discarding of samples with the application of time‐to‐independence filters. This is supported by the fact that the most social of the carnivores sampled (the spotted hyena) had the greatest cumulative loss of images among members of the carnivore guild (Table 1).

Species that lost the least amount of data in our study (the carnivores) are not only typically rare (so fewer individuals are recorded at longer intervals), but also share a similar behavior in actively searching for prey across the landscape (Estes, 2012), which likely influences their capture rate on camera traps. This contrasts with the typically more social, abundant, and patch‐specific foraging herbivores. Thus, the latter would be more likely to be represented in repeated captures on a camera trap operating in their foraging patch, while carnivores are more likely to move through a patch quickly and so accumulate fewer images within a specified time. This would exert an asymmetrical effect of time‐to‐independence filtering on these guilds, based on their life history characteristics. Thus, not only is it important to recognize that time‐to‐independence filtering alters our estimation of animal activity, but also which species or guilds of species are more or less at risk of the biases associated with time to independence. The ability to generalize our observation that estimates of some species' activity patterns may be more vulnerable than others to the application of time‐to‐independence filters needs to be tested with data from additional species, and including rare, social herbivores (with small sample sizes) and carnivores with large sample sizes. Based on these guild‐level differences in the effect of applying time to independence (differential loss of sample sizes and changes in estimated activity patterns), the use of time‐to‐independence filters in comparative activity studies of, for example, predators and prey, will lead to misleading outcomes.

These findings are also important when estimating the activity patterns of rare species, which are less frequently captured on camera traps (Lama et al., 2019). Although our results suggest that for these species, discarding data (through the application of a time‐to‐independence filter) will have a smaller effect on the proportion of available data, this may still lead to inadequate sample sizes for estimating activity patterns. This would be particularly important if discarding of activity events leads to the misclassification of activity patterns (e.g., diurnal vs. crepuscular), and hence a misunderstanding of the species' ecology, resource use patterns, and response to environmental pressures.

In many camera trap studies, “burst images” are used on the initial trigger of motion to capture additional images to improve species identifications (Wearn & Glover‐Kapfer, 2017). These burst images are user‐defined, not a true sample of animal activity, and artificially increase activity density. This means that data from these initial image bursts cannot be used to estimate activity and must be discarded from activity pattern analyses. Thus, for studies where these burst images are not specifically needed for individual identification, we suggest this setting should not be used, thus allowing for the recording of activity in a more continuous fashion.

The use of time‐to‐independence filters in studies that estimate animal activity patterns is therefore challenged under the principle that activity is a continuous state, and all records of activity of the same individual (or species) represent meaningful information. Thus, camera trap images of the same individual or group are not pseudoreplicates (sensu Hurlbert, 1984), but rather valid records of animal activity. The question arises as to whether the use of time to independence in published studies of activity patterns led to biases in the findings of these studies? A sample of such studies shows a variable approach (Figure 1) to the application of time to independence. Some studies use lengthy intervals of time to independence, ranging from 30 to 60 min (e.g., Farris et al., 2015; Foster et al., 2013; Santos et al., 2019; Tambling et al., 2015). This may have biased their results, but the nature of these effects is unknown. Studies contrasting prey and predator activity (e.g., Foster et al., 2013; Tambling et al., 2015; Smith et al., 2020; Zaman et al., 2022) that apply time‐to‐independence filters may need to be revisited. This is due to the demonstrated differences in the effects of time to independence between these two guilds. A similar effect is likely present among comparisons of activity patterns of rare and abundant species, in, for example, studies on competition (c.f. Santos et al., 2019). Furthermore, the variable and arbitrary application of the time to independence across these studies means that the derived estimates of activity patterns cannot be meaningfully compared between studies.

## 5 CONCLUSION

The use of time‐to‐independence filters in camera trap studies that describe the activity patterns of free‐ranging animals is conceptually not justified, discards valuable data, and biases our understanding of the estimated activity patterns of animals. This may lead to incorrect inferences being drawn from such studies, although the extent and nature of these are currently unknown. The convention within the published literature of using lengthy periods of time to independence (often exceeding 30 min) for such studies is therefore challenged.

Studies estimating activity patterns from camera trap data should not discard activity data by applying time to independence. Alternatively, such studies should specifically test the effects of applying time to independence on estimated activity patterns and how these estimates may respond to factors that influence activity. This will lead to a more realistic description of the activity patterns of animals based on camera trap data, allow comparisons with activity patterns derived using other approaches, and generate greater confidence in our understanding of the factors that influence these activity patterns.

## AUTHOR CONTRIBUTIONS

**Christopher Peral:** Conceptualization (equal); formal analysis (lead); methodology (equal); visualization (equal); writing – original draft (equal); writing – review and editing (equal). **Marietjie Landman:** Conceptualization (equal); formal analysis (supporting); methodology (equal); supervision (equal); visualization (equal); writing – original draft (equal); writing – review and editing (equal). **Graham I. H. Kerley:** Conceptualization (lead); methodology (equal); supervision (lead); writing – original draft (equal); writing – review and editing (equal).

## CONFLICT OF INTEREST

The authors have no conflicts of interest to declare.

## Supporting information


Table S1‐S2

## Data Availability

The dataset of this study is available on https://lila.science/datasets/snapshot‐serengeti.
