# Supplementary material for: The inappropriate use of time‐to‐independence biases estimates of activity patterns of free‐ranging mammals derived from camera traps
Source: Ecol Evol. 2022 Oct 13;12(10):e9408. doi: 10.1002/ece3.9408 (PMC9596328; doi:10.1002/ece3.9408)
Supplement: Supplementary file 1 — Table S1‐S2 [file ECE3-12-e9408-s001.docx]

**SUPPORTING INFORMATION**

Supporting Information for Peral et al: The inappropriate use of time-to-independence influences estimates of activity patterns of free-ranging mammals derived from camera traps

TABLE S1 List of open-access publications, extracted from Web of Science (1998-2021), that use camera traps to describe animal activity patterns, indicating whether or not time-to-independence (TTI) was applied, and if so, the duration of this time-to independence.

| **Reference** | **TTI?** | **Time** |
| --- | --- | --- |
| Ramesh, Tharmalingam; Downs, Colleen T.; 2013. Impact Of Farmland Use On Population Density And Activity Patterns Of Serval In South Africa. Journal Of Mammalogy 94: 1460-1470. DOI: 10.1644/13-Mamm-A-063.1. | Yes | 1 Min |
| Karanth, K. Ullas; Srivathsa, Arjun; Vasudev, Divya; Puri, Mahi; Parameshwaran, Ravishankar; Kumar, N. Samba; 2017. Spatio-Temporal Interactions Facilitate Large Carnivore Sympatry Across A Resource Gradient. Proceedings Of The Royal Society B-Biological Sciences 284. DOI: 10.1098/Rspb.2016.1860. | Yes | 1 Min |
| Kays, Roland; Costello, Robert; Forrester, Tavis; Baker, Megan C.; Parsons, Arielle W.; Kalies, Elizabeth L.; Hess, George; Millspaugh, Joshua J.; McShea, William; 2015. Cats Are Rare Where Coyotes Roam. Journal Of Mammalogy 96: 981-987. DOI: 10.1093/Jmammal/Gyv100. | Yes | 1 Min |
| Unal, Y.; Eryilmaz, A.; 2020. Jungle Cat (Felis Chaus Schreber, 1777) Population Density Estimates, Activity Pattern And Spatiotemporal Interactions With Humans And Other Wildlife Species In Turkey. Applied Ecology And Environmental Research 18: 5873-5890. DOI: 10.15666/Aeer/1804_58735890. | Yes | 5 Min |
| Rottstock, Thomas; Goettert, Thomas; Zeller, Ulrich; 2020. Relatively Undisturbed African Savannas - An Important Reference For Assessing Wildlife Responses To Livestock Grazing Systems In European Rangelands. Global Ecology And Conservation 23. DOI: 10.1016/J.Gecco.2020.E01124. | Yes | 5 Min |
| Diete, Rebecca L.; Meek, Paul D.; Dickman, Christopher R.; Lisle, Allan; Leung, Luke K. -P.; 2017. Diel Activity Patterns Of Northern Australian Small Mammals: Variation, Fixity, And Plasticity. Journal Of Mammalogy 98: 848-857. DOI: 10.1093/Jmammal/Gyx003. | Yes | 5 Min |
| Carricondo-Sanchez, David; Odden, Morten; Kulkarni, Abhijeet; Vanak, Abi Tamim; 2019. Scale-Dependent Strategies For Coexistence Of Mesocarnivores In Human-Dominated Landscapes. Biotropica 51: 781-791. DOI: 10.1111/Btp.12705. | Yes | 10 Min |
| Richardson, Matthew L.; 2017. Daily And Monthly Activity Of Brown Bears (*Ursus arctos*) Near A Proposed Industrial Project In Coastal British Columbia. Western North American Naturalist 77: 118-123. DOI: 10.3398/064.077.0113. | Yes | 10 Min |
| Allen, M. L.; Sibarani, M. C.; Utoyo, L.; Krofel, M.; 2020. Terrestrial Mammal Community Richness And Temporal Overlap Between Tigers And Other Carnivores In Bukit Barisan Selatan National Park, Sumatra. Animal Biodiversity And Conservation 43: 97-107. DOI: 10.32800/Abc.2020.43.0097. | Yes | 30 Min |
| Luo, Gai; Yang, Chuangming; Zhou, Huaming; Seitz, Michael; Wu, Yongjie; Ran, Jianghong; 2019. Habitat Use And Diel Activity Pattern Of The Tibetan Snowcock (*Tetraogallust tibetanus*): A Case Study Using Camera Traps For Surveying High-Elevation Bird Species. Avian Research 10. DOI: 10.1186/S40657-019-0144-Y. | Yes | 30 Min |
| Zou, Fasheng; Zhang, Qiang; Zhang, Min; Lee, Myung-Bok; Wang, Xincai; Gong, Yuening; Yang, Changteng; 2019. Temporal Patterns Of Three Sympatric Pheasant Species In The Nanling Mountains: N-Mixture Modeling Applied To Detect Abundance. Avian Research 10. DOI: 10.1186/S40657-019-0181-6. | Yes | 30 Min |
| Wang, Yiwei; Allen, Maximilian L.; Wilmers, Christopher C.; 2015. Mesopredator Spatial And Temporal Responses To Large Predators And Human Development In The Santa Cruz Mountains Of California. Biological Conservation 190: 23-33. DOI: 10.1016/J.Biocon.2015.05.007. | Yes | 30 Min |
| Zhang, Jindong; Hull, Vanessa; Ouyang, Zhiyun; Li, Rengui; Connor, Thomas; Yang, Hongbo; Zhang, Zejun; Silet, Brad; Zhang, Hemin; Liu, Jianguo; 2017. Divergent Responses Of Sympatric Species To Livestock Encroachment At Fine Spatiotemporal Scales. Biological Conservation 209: 119-129. DOI: 10.1016/J.Biocon.2017.02.014. | Yes | 30 Min |
| Osorio, Christian; Munoz, Ana; Guarda, Nicolas; Bonacic, Cristian; Kelly, Marcella; 2020. Exotic Prey Facilitate Coexistence Between Pumas And Culpeo Foxes In The Andes Of Central Chile. Diversity-Basel 12. DOI: 10.3390/D12090317. | Yes | 30 Min |
| Vilella, Marc; Ferrandiz-Rovira, Mariona; Sayol, Ferran; 2020. Coexistence Of Predators In Time: Effects Of Season And Prey Availability On Species Activity Within A Mediterranean Carnivore Guild. Ecology And Evolution 10: 11408-11422. DOI: 10.1002/Ece3.6778. | Yes | 30 Min |
| Easter, Tara; Bouley, Paola; Carter, Neil; 2020. Intraguild Dynamics Of Understudied Carnivores In A Human-Altered Landscape. Ecology And Evolution 10: 5476-5488. DOI: 10.1002/Ece3.6290. | Yes | 30 Min |
| Sasidhran, Selvadurai; Adila, Nurfatin; Hamdan, Mohd Saifulnizam; Samantha, Liza D.; Aziz, Najjib; Kamarudin, Norizah; Puan, Chong Leong; Turner, Edgar; Azhar, Badrul; 2016. Habitat Occupancy Patterns And Activity Rate Of Native Mammals In Tropical Fragmented Peat Swamp Reserves In Peninsular Malaysia. Forest Ecology And Management 363: 140-148. DOI: 10.1016/J.Foreco.2015.12.037. | Yes | 30 Min |
| Chatterjee, Nilanjan; Nigam, Parag; Habib, Bilal; 2020. Population Estimate, Habitat-Use And Activity Patterns Of The Honey Badger In A Dry-Deciduous Forest Of Central India. Frontiers In Ecology And Evolution 8. DOI: 10.3389/Fevo.2020.585256. | Yes | 30 Min |
| Ota, Ayana; Takagi, Etsuro; Yasuda, Masatoshi; Hashim, Mazlan; Hosaka, Tetsuro; Numata, Shinya; 2019. Effects Of Nonlethal Tourist Activity On The Diel Activity Patterns Of Mammals In A National Park In Peninsular Malaysia. Global Ecology And Conservation 20. DOI: 10.1016/J.Gecco.2019.E00772. | Yes | 30 Min |
| Fan, Fan; Bu, Hongliang; McShea, William J.; Shen, Xiaoli; Li, Binbin V.; Li, Sheng; 2020. Seasonal Habitat Use And Activity Patterns Of Blood Pheasant *Ithaginis Cruentusbe* In The Presence Of Free-Ranging Livestock. Global Ecology And Conservation 23. DOI: 10.1016/J.Gecco.2020.E01155. | Yes | 30 Min |
| Tian, Cheng; Zhang, Yu-Yang; Liu, Zheng-Xiao; Dayananda, Buddhi; Fu, Xiao-Bo; Yuan, Dan; Tu, Zheng-Bin; Luo, Chun-Ping; Li, Jun-Qing; 2020. Temporal Niche Patterns Of Large Mammals In Wanglang National Nature Reserve, China. Global Ecology And Conservation 22. DOI: 10.1016/J.Gecco.2020.E01015. | Yes | 30 Min |
| Bersacola, Elena; Sastramidjaja, Wiwit; Rayadin, Yaya; Macdonald, David; Cheyne, Susan M.; 2019. Occupancy Patterns Of Ungulates And Pig-Tailed Macaques Across Regenerating And Anthropogenic Forests On Borneo. Hystrix-Italian Journal Of Mammalogy 30: 126-133. DOI: 10.4404/Hystrix-00177-2019. | Yes | 30 Min |
| Miller, Jennifer R. B.; Pitman, Ross T.; Mann, Gareth K. H.; Fuller, Angela K.; Balme, Guy A.; 2018. Lions And Leopards Coexist Without Spatial, Temporal Or Demographic Effects Of Interspecific Competition. Journal Of Animal Ecology 87: 1709-1726. DOI: 10.1111/1365-2656.12883. | Yes | 30 Min |
| Xue Yadong; Li Jia; Sagen Guli; Zhang Yu; Dai Yunchuan; Li Diqiang; 2018. Activity Patterns And Resource Partitioning: Seven Species At Watering Sites In The Altun Mountains, China. Journal Of Arid Land 10: 959-967. DOI: 10.1007/S40333-018-0028-8. | Yes | 30 Min |
| Lee, Hwa-Jin; Ha, Jeong-Wook; Park, Seong-Joon; Kim, Woo-Yuel; Cha, Jin-Yeol; Park, Jin-Young; Choi, Seung-Se; Chung, Chul-Un; Oh, Hong-Shik; 2019. A Study On The Analysis Of Mammals' Activity Patterns And The Effect Of Human Hiker Interference Using Camera Trapping. Journal Of Asia-Pacific Biodiversity 12: 57-62. DOI: 10.1016/J.Japb.2018.11.009. | Yes | 30 Min |
| Mugerwa, Badru; du Preez, Byron; Tallents, Lucy A.; Loveridge, Andrew J.; Macdonald, David W.; 2017. Increased Foraging Success Or Competitor Avoidance? Diel Activity Of Sympatric Large Carnivores. Journal Of Mammalogy 98: 1443-1452. DOI: 10.1093/Jmammal/Gyx090. | Yes | 30 Min |
| Blake, John G.; Mosquera, Diego; Loiselle, Bette A.; Swing, Kelly; Guerra, Jaime; Romo, David; 2016. Spatial And Temporal Activity Patterns Of Ocelots *Leopardus Pardalis* In Lowland Forest Of Eastern Ecuador. Journal Of Mammalogy 97: 455-463. DOI: 10.1093/Jmammal/Gyv190. | Yes | 30 Min |
| Botts, Ryan T.; Eppert, Amy A.; Wiegman, Timothy J.; Rodriguez, Abner; Blankenship, Steven R.; Asselin, Ellen M.; Garley, Wyatt M.; Wagner, Abigail P.; Ullrich, Sierra E.; Allen, Gabrielle R.; Mooring, Michael S.; 2020. Circadian Activity Patterns Of Mammalian Predators And Prey In Costa Rica. Journal Of Mammalogy 101: 1313-1331. DOI: 10.1093/Jmammal/Gyaa103. | Yes | 30 Min |
| Linkie, M.; Ridout, M. S.; 2011. Assessing Tiger-Prey Interactions In Sumatran Rainforests. Journal Of Zoology 284: 224-229. DOI: 10.1111/J.1469-7998.2011.00801.X. | Yes | 30 Min |
| Bischof, R.; Ali, H.; Kabir, M.; Hameed, S.; Nawaz, M. A.; 2014. Being The Underdog: An Elusive Small Carnivore Uses Space With Prey And Time Without Enemies. Journal Of Zoology 293: 40-48. DOI: 10.1111/Jzo.12100. | Yes | 30 Min |
| Zanni, Michele; Brivio, Francesca; Grignolio, Stefano; Apollonio, Marco; 201. Estimation Of Spatial And Temporal Overlap In Three Ungulate Species In A Mediterranean Environment. Mammal Research. DOI: 10.1007/S13364-020-00548-1. | Yes | 30 Min |
| Rasphone, Akchousanh; Kamler, Jan F.; Macdonald, David W.; 2020. Temporal Partitioning By Felids, Dholes And Their Potential Prey In Northern Laos. Mammal Research 65: 679-689. DOI: 10.1007/S13364-020-00524-9. | Yes | 30 Min |
| Mori, Emiliano; Andreoni, Alley; Cecere, Francesco; Magi, Matteo; Lazzeri, Lorenzo; 2020. Patterns Of Activity Rhythms Of Invasive Coypus *myocastor Coypus* inferred Through Camera-Trapping. Mammalian Biology 100: 591-599. DOI: 10.1007/S42991-020-00052-8. | Yes | 30 Min |
| Nenov, Dimitar I.; Zlatanova, Diana P.; Stoynov, Emilian H.; Peshev, Hristo V.; Grozdanov, Atanas P.; 2018. Feeding Site Usage By Griffon Vultures (*Gyps fulvus*) In Bulgaria Revealed By Camera Traps. Nature Conservation Research 3: 2-12. DOI: 10.24189/Ncr.2018.020. | Yes | 30 Min |
| Sidorchuk, Natalia V.; Rozhnov, Viatcheslav V.; 2018. Daily Activity Of The European Badger (*Meles meles*, Mustelidae, Carnivora) On Setts In Darwin Reserve And Meschera National Park (Russia) In Summer And Autumn. Nature Conservation Research 3: 47-56. DOI: 10.24189/Ncr.2018.032. | Yes | 30 Min |
| Li, Jia; Xue, Yadong; Zhang, Yu; Dong, Wei; Shan, Guoyu; Sun, Ruiqian; Hacker, Charlotte; Wu, Bo; Li, Diqiang; 2020. Spatial And Temporal Activity Patterns Of Golden Takin (*Budorcas taxicolor bedfordi)* Recorded By Camera Trapping. Peerj 8. DOI: 10.7717/Peerj.10353. | Yes | 30 Min |
| Caruso, Nicolas; Lucherini, Mauro; Fortin, Daniel; Casanave, Emma B.; 2016. Species-Specific Responses Of Carnivores To Human-Induced Landscape Changes In Central Argentina. Plos One 11. DOI: 10.1371/Journal.Pone.0150488. | Yes | 30 Min |
| Caruso, Nicolas; Valenzuela, Alejandro E. J.; Burdett, Christopher L.; Luengos Vidal, Estela M.; Birochio, Diego; Casanave, Emma B.; 2018. Summer Habitat Use And Activity Patterns Of Wild Boar *Sus Scrofa* In Rangelands Of Central Argentina. Plos One 13. DOI: 10.1371/Journal.Pone.0206513. | Yes | 30 Min |
| Chaudhary, Rohit; Zehra, Nazneen; Musavi, Azra; Khan, Jamal Ahmad; 2020. Spatio-Temporal Partitioning And Coexistence Between Leopard (*Panthera pardus fusca*) And Asiatic Lion (*Panthera leo persica*) In Gir Protected Area, Gujarat, India. Plos One 15. DOI: 10.1371/Journal.Pone.0229045. | Yes | 30 Min |
| Thapa, Kanchan; Kelly, Marcella J.; Pradhan, Narendra Man Babu; 2019. Elephant (*Elephas Maximus*) Temporal Activity, Distribution, And Habitat Use Patterns On The Tiger'S Forgotten Trails Across The Seasonally Dry, Subtropical, Hilly Churia Forests Of Nepal. Plos One 14. DOI: 10.1371/Journal.Pone.0216504. | Yes | 30 Min |
| de Oliveira, Emila Silveira; Fontoura Rodrigues, Manoel Ludwig; Severo, Magnus Machado; dos Santos, Tiago Gomes; Kasper, Carlos Benhur; 2020. Who'S Afraid Of The Big Bad Boar? Assessing The Effect Of Wild Boar Presence On The Occurrence And Activity Patterns Of Other Mammals. Plos One 15. DOI: 10.1371/Journal.Pone.0235312. | Yes | 30 Min |
| Edwards, Sarah; Noack, Jenny; Heyns, Louis; Rodenwoldt, Diethardt; 2015. Are Camera Traps A Reliable Method For Estimating Activity Patterns? A Case Study Comparing Technologies For Estimating Brown Hyaena Activity Curves. Remote Sensing In Ecology And Conservation. DOI: 10.1002/Rse2.175. | Yes | 30 Min |
| Blake, John G.; Mosquera, Diego; Guerra, Jaime; Loiselle, Bette A.; Romo, David; Swing, Kelly; 2014. Yasuni - A Hotspot For Jaguars *Panthera Onca* (Carnivora: Felidae)? Camera-Traps And Jaguar Activity At Tiputini Biodiversity Station, Ecuador. Revista De Biologia Tropical 62: 689-698. DOI: 10.15517/Rbt.V62I2.11115. | Yes | 30 Min |
| Mooring, Michael S.; Eppert, Amy A.; Botts, Ryan T.; 2020. Natural Selection Of Melanism In Costa Rican Jaguar And Oncilla: A Test Of Gloger'S Rule And The Temporal Segregation Hypothesis. Tropical Conservation Science 13. DOI: 10.1177/1940082920910364. | Yes | 30 Min |
| Botts, Ryan T.; Eppert, Amy A.; Wiegman, Timothy J.; Blankenship, Steven R.; Rodriguez, Abner; Wagner, Abigail P.; Ullrich, Sierra E.; Allen, Gabrielle R.; Garley, Wyatt M.; Asselin, Ellen M.; Mooring, Michael S.; 2020. Does Moonlight Increase Predation Risk For Elusive Mammals In Costa Rica? Tropical Conservation Science 13. DOI: 10.1177/1940082920952405. | Yes | 30 Min |
| Dou, Hailong; Yang, Haitao; Smith, James L. D.; Feng, Limin; Wang, Tianming; Ge, Jianping; 2019. Prey Selection Of Amur Tigers In Relation To The Spatiotemporal Overlap With Prey Across The Sino-Russian Border. Wildlife Biology. DOI: 10.2981/Wlb.00508. | Yes | 30 Min |
| Parres, Aida; Palazon, Santiago; Afonso, Ivan; Quenette, Pierre-Yves; Batet, Antoni; Camarra, Jean-Jacques; Garreta, Xavier; Goncalves, Salvador; Guillen, Jordi; Mir, Sergio; Jato, Ramon; Rodriguez, Joan; Sentilles, Jerome; Xicola, Laura; Melero, Yolanda; 2020. Activity Patterns In The Reintroduced Pyrenean Brown Bear Population. Mammal Research 65: 435-444. DOI: 10.1007/S13364-020-00507-W. | Yes | 30 Min |
| Zhao, Guojing; Yang, Haitao; Xie, Bing; Gong, Yinan; Ge, Jianping; Feng, Limin; 2020. Spatio-Temporal Coexistence Of Sympatric Mesocarnivores With A Single Apex Carnivore In A Fine-Scale Landscape. Global Ecology And Conservation 21. DOI: 10.1016/J.Gecco.2019.E00897. | Yes | 30 Min |
| Maria Avila-Najera, Dulce; Antonio Lazcano-Barrero, Marco; Chavez, Cuauhtemoc; Perez-Elizalde, Sergio; Tigar, Barbara; David Mendoza, German; 2019. Habitat Use Of Jaguar (*Panthera onca*) In A Tropical Forest In Northern Quintana Roo, Mexico. Revista Mexicana De Biodiversidad 90. DOI: 10.22201/Ib.20078706E.2019.90.2186. | Yes | 30 Min |
| Cruz, Paula; Eugenia Iezzi, Maria; De Angelo, Carlos; Varela, Diego; Di Bitetti, Mario S.; Pavioio, Agustin; 2018. Effects Of Human Impacts On Habitat Use, Activity Patterns And Ecological Relationships Among Medium And Small Felids Of The Atlantic Forest. Plos One 13. DOI: 10.1371/Journal.Pone.0200806. | Yes | 60 Min |
| Tan, Chia L.; Yang, Yeqin; Niu, Kefeng; 2013. Into The Night: Camera Traps Reveal Nocturnal Activity In A Presumptive Diurnal Primate, *Rhinopithecus Brelichi*. Primates 54: 1-6. DOI: 10.1007/S10329-012-0318-2. | Yes | 60 Min |
| Beirne, Christopher; Pillco-Huarcaya, Ruthmery; Jennifer Serrano-Rojas, Shirley; Whitworth, Andrew; 2017. Terrestrial Camera Traps: Essential Tool For The Detection And Future Monitoring Of The Critically Endangered Sira *curassow pauxi koepckeae*. Endangered Species Research 32: 145-152. DOI: 10.3354/Esr00802. | Yes | 60 Min |
| Magalhaes, Laura Martins; Srbek-Artujo, Ana Carolina; 2019. Plasticity In The Timing Of Activity In The Red-Rumped Agouti, *Dasyprocta leporina* (Mammalia: Rodentia), In The Atlantic Forest Of Southeastern Brazil. Biota Neotropica 19. DOI: 10.1590/1676-0611-Bn-2018-0625. | Yes | 60 Min |
| Kuhnen, Vanessa V.; De Lima, R. E. M.; Santos, J. F.; Machado Filho, L. C. P.; 2013. Habitat Use And Circadian Pattern Of Solitary Tinamou *Tinamus Solitarius* In A Southern Brazilian Atlantic Rainforest. Bird Conservation International 23: 78-82. DOI: 10.1017/S0959270912000147. | Yes | 60 Min |
| Graipel, M. E.; Oliveira-Santos, L. G. R.; Goulart, F. V. B.; Tortato, M. A.; Miller, P. R. M.; Caceres, N. C.; 2014. The Role Of Melanism In Oncillas On The Temporal Segregation Of Nocturnal Activity. Brazilian Journal Of Biology 74: 142-145. DOI: 10.1590/1519-6984.14312. | Yes | 60 Min |
| Leuchtenberger, C.; de Oliveira, E. S.; Cariolatto, L. P.; Kasper, C. B.; 2018. Activity Pattern Of Medium And Large Sized Mammals And Density Estimates Of *Cuniculus Paca* (Rodentia: Cuniculidae) In The Brazilian Pampa. Brazilian Journal Of Biology 78: 697-705. DOI: 10.1590/1519-6984.174403. | Yes | 60 Min |
| Mills, David R.; San, Emmanuel Do Linh; Robinson, Hugh; Isoke, Sam; Slotow, Rob; Hunter, Luke; 2019. Competition And Specialization In An African Forest Carnivore Community. Ecology And Evolution 9: 10092-10108. DOI: 10.1002/Ece3.5391. | Yes | 60 Min |
| Lara-Diaz, Nalleli E.; Coronel-Arellano, Heli; Lopez-Gonzalez, Carlos A.; Sanchez-Rojas, Gerardo; Esteban Martinez-Gomez, Juan; 2018. Activity And Resource Selection Of A Threatened Carnivore: The Case Of Black Bears In Northwestern Mexico. Ecosphere 9. DOI: 10.1002/Ecs2.1923. | Yes | 60 Min |
| Brzezinski, Marcin; Jedlikowski, Jan; Komar, Ewa; 2019. Space Use, Habitat Selection And Daily Activity Of Water Voles *Arvicola amphibius* Co-Occurring With The Invasive American Mink *Neovison vison*. Folia Zoologica 68: 21-28. DOI: 10.25225/Fozo.040.2019. | Yes | 60 Min |
| Massara, Rodrigo L.; Paschoal, Ana Maria O.; Bailey, Larissa L.; Doherty, Paul F., Jr.; Chiarello, Adriano G.; 2016. Ecological Interactions Between Ocelots And Sympatric Mesocarnivores In Protected Areas Of The Atlantic Forest, Southeastern Brazil. Journal Of Mammalogy 97: 1634-1644. DOI: 10.1093/Jmammal/Gyw129. | Yes | 60 Min |
| Fonturbel, Francisco E.; Candia, Alina B.; Botto-Mahan, Carezza; 2014. Nocturnal Activity Patterns Of The Monito Del Monte (*Dromiciops gliroides*) In Native And Exotic Habitats. Journal Of Mammalogy 95: 1199-1206. DOI: 10.1644/13-Mamm-A-304. | Yes | 60 Min |
| Cid, Bruno; Oliveira-Santos, Luiz Gustavo R.; Mourao, Guilherme; 2015. The Relationship Between External Temperature And Daily Activity In A Large Rodent (*Dasyprocta azarae*) In The Brazilian Pantanal. Journal Of Tropical Ecology 31: 469-472. DOI: 10.1017/S0266467415000309. | Yes | 60 Min |
| Gouda, Sushanto; Chauhan, Netrapal Singh; Sethy, Janmejay; Sahu, Hemanta Kumar; 2020. Daily Activity Pattern Of Malayan Sun Bear In Dampa Tiger Reserve, Mizoram, India. Journal Of Wildlife And Biodiversity 4: 56-64. DOI: 10.22120/Jwb.2020.117400.1103. | Yes | 60 Min |
| Harmsen, Bart J.; Sanchez, Emma; Figueroa, Omar A.; Gutierrez, Said M.; Doncaster, C. Patrick; Foster, Rebecca J.; 2019. Ecology Of A Versatile Canid In The Neotropics: Gray Foxes (*Urocyon cinereoargenteus*) In Belize, Central America. Mammal Research 64: 319-332. DOI: 10.1007/S13364-018-00413-2. | Yes | 60 Min |
| Mos, Jeroen; Hofmeester, Tim Ragnvald; 2020. The Mostela: An Adjusted Camera Trapping Device As A Promising Non-Invasive Tool To Study And Monitor Small Mustelids. Mammal Research 65: 843-853. DOI: 10.1007/S13364-020-00513-Y. | Yes | 60 Min |
| de Oliveira, Marcio Leite; de Faria Peres, Pedro Henrique; Vogliotti, Alexandre; Grotta-Neto, Francisco; Koester de Azevedo, Allyson Diaz; Cerveira, Josi Fernanda; do Nascimento, Guilherme Batista; Peruzzi, Nelson Jose; Carranza, Juan; Barbanti Duarte, Jose Mauricio; 2016. Phylogenetic Signal In The Circadian Rhythm Of Morphologically Convergent Species Of Neotropical Deer. Mammalian Biology 81: 281-289. DOI: 10.1016/J.Mambio.2016.01.004. | Yes | 60 Min |
| Carvalho, William Douglas; Rosalino, Luis Miguel; Godoy, Maira Sant'Ana M.; Giorgete, Marilia F.; Adania, Cristina Harumi; Esberard, Carlos E. Lustosa; 2019. Temporal Activity Of Rural Free-Ranging Dogs: Implications For The Predator And Prey Species In The Brazilian Atlantic Forest. Neobiota : 55-74. DOI: 10.3897/Neobiota.45.30645. | Yes | 60 Min |
| Dobbins, Michael T.; Steinberg, Michael K.; Broadbent, Eben N.; Ryan, Sadie J.; 2018. Habitat Use, Activity Patterns And Human Interactions With Jaguars *Panthera onca* In Southern Belize. Oryx 52: 276-281. DOI: 10.1017/S0030605317000308. | Yes | 60 Min |
| Caravaggi, Anthony; Gatta, Maria; Vallely, Marie-Claire; Hogg, Kayleigh; Freeman, Marianne; Fadaei, Erfan; Dick, Jaimie T. A.; Montgomery, W. Ian; Reid, Neil; Tosh, David G.; 2018. Seasonal And Predator-Prey Effects On Circadian Activity Of Free-Ranging Mammals Revealed By Camera Traps. Peerj 6. DOI: 10.7717/Peerj.5827. | Yes | 60 Min |
| Gardner, Penny C.; Goossens, Benoit; Wern, Jocelyn Goon Ee; Kretzschmar, Petra; Bohm, Torsten; Vaughan, Ian P.; 2018. Spatial And Temporal Behavioural Responses Of Wild Cattle To Tropical Forest Degradation. Plos One 13. DOI: 10.1371/Journal.Pone.0195444. | Yes | 60 Min |
| Perez-Irineo, Gabriela; Santos-Moreno, Antonio; 2014. Density, Distribution, And Activity Of The Ocelot *Leopardus Pardalis* (Carnivora: Felidae) In Southeast Mexican Rainforests. Revista De Biologia Tropical 62: 1421-1432. DOI: 10.15517/Rbt.V62I4.12941. | Yes | 60 Min |
| Ancrenaz, Marc; Sollmann, Rahel; Meijaard, Erik; Hearn, Andrew J.; Ross, Joanna; Samejima, Hiromitsu; Loken, Brent; Cheyne, Susan M.; Stark, Danica J.; Gardner, Penny C.; Goossens, Benoit; Mohamed, Azlan; Bohm, Torsten; Matsuda, Ikki; Nakabayasi, Miyabi; Lee, Shan Khee; Bernard, Henry; Brodie, Jedediah; Wich, Serge; Fredriksson, Gabriella; Hanya, Goro; Harrison, Mark E.; Kanamori, Tomoko; Kretzschmar, Petra; Macdonald, David W.; Riger, Peter; Spehar, Stephanie; Ambu, Laurentius N.; Wilting, Andreas; 2014. Coming Down From The Trees: Is Terrestrial Activity In Bornean Orangutans Natural Or Disturbance Driven? Scientific Reports 4. DOI:10.1038/Srep04024. | Yes | 60 Min |
| Paulo Carbajal-Borges, Juan; Godinez-Gomez, Oscar; Mendoza, Eduardo; 2014. Density, Abundance And Activity Patterns Of The Endangered *Tapirus bairdii* In One Of Its Last Strongholds In Southern Mexico. Tropical Conservation Science 7: 100-114. DOI: 10.1177/194008291400700102. | Yes | 60 Min |
| Oliveira-Santos, Luiz Gustavo R.; Graipel, Mauricio E.; Tortato, Marcos A.; Zucco, Carlos A.; Caceres, Nilton C.; Goulart, Fernando V. B.; 2012. Abundance Changes And Activity Flexibility Of The Oncilla, *Leopardus tigrinus* (Carnivora: Felidae), Appear To Reflect Avoidance Of Conflict. Zoologia 29: 115-120. DOI: 10.1590/S1984-46702012000200003. | Yes | 60 Min |
| Borchard, P.; 2013. Using Camera - Trap Data To Model Habitat Use By Wombats And Cattle In Australian Riparian Ecosystems. Applied Ecology And Environmental Research 11: 21-33. DOI: 10.15666/Aeer/1101_021033. | Yes | 60 Min |
| Hernandez-Perez, Edwin L.; Castillo-Vela, Guillermo; Garcia-Marmolejo, Gabriela; Hidalgo-Mihart, Mircea; Contreras-Moreno, Fernando M.; Jesus-de La Cruz, Alejandro; Juarez-Lopez, Rugieri; Reyna-Hurtado, Rafael; 2020. Ecological Relationships Between Collared Peccaries And Feral Pigs In Southern Mexico: Evidence For Niche Partitioning?. Revista Mexicana De Biodiversidad 91. DOI: 10.22201/Ib.20078706E.2020.91.2977. | Yes | 60 Min |
| Webb, Edward L.; Choo, Yan Ru; Kudavidanage, Enoka P.; Amarasinghe, Thakshila Ravindra; Bandara, Udamulle Gedara Sumith Indika; Wanninay, Wanninayaka Aarahchilage Charitha Lakmali; Ravindrakumar, Piyal; Nimalrathna, Thilina Sudarshana; Liang, Song Horng; Chua, Marcus Aik Hwee; 2020. Leopard Activity Patterns In A Small Montane Protected Area Highlight The Need For Integrated, Collaborative Landscape Conservation. Global Ecology And Conservation 23. DOI: 10.1016/J.Gecco.2020.E01182. | Yes | 60 Min |
| Di Bitetti, Mario S.; Di Blanco, Yamil E.; Pereira, Javier A.; Paviolo, Agustin; Jimenez Perez, Ignacio; 2009. Time Partitioning Favors The Coexistence Of Sympatric Crab-Eating Foxes (*Cerdocyon* *thous*) And Pampas Foxes (*Lycalopex Gymnocercus*). Journal Of Mammalogy 90: 479-490. DOI: 10.1644/08-Mamm-A-113.1. | Yes | 12 Hrs |
| Rorabaugh, James C.; Schipper, Jan; Avila-Villegas, Sergio; Lamberton-Moreno, Jessica A.; Flood, Timothy; 2020. Ecology Of An Ocelot Population At The Northern Edge Of The Species' Distribution In Northern Sonora, Mexico. Peerj 8. DOI: 10.7717/Peerj.8414. | Yes | 15 Min |
| Serna-Lagunes, R.; Alvarez-Oseguera, L. R.; Avila-Najera, D. M.; Leyva-Ovalle, O. R.; Andres-Meza, P.; Tigar, B.; 2019. Temporal Overlap In The Activity Of *Lynx Rufu*s And *Canis Latrans* And Their Potential Prey In The Pico De Orizaba National Park, Mexico. Animal Biodiversity And Conservation 42: 153-161. DOI: 10.32800/Abc.2019.42.0153. | Yes | 180 Min |
| Barrueto, Mirjam; Ford, Adam T.; Clevenger, Anthony P.; 2014. Anthropogenic Effects On Activity Patterns Of Wildlife At Crossing Structures. Ecosphere 5. DOI: 10.1890/Es13-00382.1. | Yes | 2 Min |
| Carrera-Trevino, Rogelio; Astudillo-Sanchez, Claudia C.; Garza-Torres, Hector A.; Martinez-Garcia, Luis; Soria-Diaz, Leroy; 2018. Temporal And Spatial Interactions Of Sympatric Mesocarnivores At A Biosphere Reserve: Coexistence Or Competition ?. Revista De Biologia Tropical 66: 996-1008. DOI: 10.15517/Rbt.V66I3.30418. | Yes | 24 Hrs |
| Ferreguetti, Atilla C.; Tomas, Walfrido M.; Bergallo, Helena G.; 2015. Density, Occupancy, And Activity Pattern Of Two Sympatric Deer (Mazama) In The Atlantic Forest, Brazil. Journal Of Mammalogy 96: 1245-1254. DOI: 10.1093/Jmammal/Gyv132. | Yes | 24 Hrs |
| Norris, Darren; Michalski, Fernanda; Peres, Carlos A.; 2010. Habitat Patch Size Modulates Terrestrial Mammal Activity Patterns In Amazonian Forest Fragments. Journal Of Mammalogy 91: 551-560. DOI: 10.1644/09-Mamm-A-199.1. | Yes | 24 Hrs |
| Porfirio, Grasiela; Foster, Vania C.; Sarmento, Pedro; Fonseca, Carlos; 2018. Camera Traps As A Tool For Carnivore Conservation In A Mosaic Of Protected Areas In The Pantanal Wetlands, Brazil. Nature Conservation Research 3: 57-67. DOI: 10.24189/Ncr.2018.035. | Yes | 24 Hrs |
| Ferreguetti, Atilla Colombo; Tomas, Walfrido M.; Bergallo, Helena G.; 2016. Density And Niche Segregation Of Two Armadillo Species (Xenarthra: Dasypodidae) In The Vale Natural Reserve, Brazil. Mammalian Biology 81: 138-145. DOI: 10.1016/J.Mambio.2015.10.007. | Yes | 24 Hrs |
| Moreira-Arce, Dario; Vergara, Pablo M.; Boutin, Stan; 2015. Diurnal Human Activity And Introduced Species Affect Occurrence Of Carnivores In A Human-Dominated Landscape. Plos One 10. DOI: 10.1371/Journal.Pone.0137854. | Yes | 24 Hrs |
| Swanson, Alexandra; Arnold, Todd; Kosmala, Margaret; Forester, James; Packer, Craig; 2016. In The Absence Of A Landscape Of Fear: How Lions, Hyenas, And Cheetahs Coexist. Ecology And Evolution 6: 8534-8545. DOI: 10.1002/Ece3.2569. | Yes | 24 Hrs |
| Ferreguetti, Atilla Colombo; Davis, Courtney Lynn; Tomas, Walfrido Moraes; Bergallo, Helena Godoy; 2018. Using Activity And Occupancy To Evaluate Niche Partitioning: The Case Of Two Peccary Species In The Atlantic Rainforest, Brazil. Hystrix-Italian Journal Of Mammalogy 29: 168-174. DOI: 10.4404/Hystrix-00068-2018. | No | None |
| Ramirez-Mejia, Andres F.; Sanchez, Francisco; 2016. Activity Patterns And Habitat Use Of Mammals In An Andean Forest And A Eucalyptus Reforestation In Colombia. Hystrix-Italian Journal Of Mammalogy 27. DOI: 10.4404/Hystrix-27.2-11319. | No | None |
| Prpic, Ana Marija; Gancevic, Pavao; Safner, Toni; Kavcic, Kresimir; Jerina, Klemen; Sprem, Nikica; 2020. Activity Patterns Of Aoudad (*Ammotragus lervia*) In A Mediterranean Habitat. Journal Of Vertebrate Biology 69. DOI: 10.25225/Jvb.20055. | No | None |
| Keten, Akif; 2016. Spatial And Temporal Distribution Of Carnivora (Mammalia) Species In Duzce Province. Kastamonu University Journal Of Forestry Faculty 16: 568-574. DOI: . | No | None |
| Nichols, Carol Anne; Alexander, Kathleen; 2018. Creeping In The Night: What Might Ecologists Be Missing? Plos One 13. DOI: 10.1371/Journal.Pone.0198277. | No | None |
| Hernandez Hernandez, Julio C.; Chavez, Cuauhtemoc; List, Rurik; 2018. Diversity And Activity Patterns Of Medium And Large Mammals In La Encrucijada Biosphere Reserve, Chiapas, Mexico. Revista De Biologia Tropical 66: 634-646. DOI: 10.15517/Rbt.V66I2.33395. | No | None |
| Stepanova, Valentina V.; Argunov, Alexandr V.; Kirillin, Ruslan A.; Okhlopkov, Innokentiy M.; 2017. Time-Study Of Moose (*Alces alces* L., 1758) Geophagia Activity In The Central Yakutia. Russian Journal Of Theriology 16: 185-190. DOI: 10.15298/Rusjtheriol.16.2.07. | No | None |
| Ikeda, Takashi; Takahashi, Hiroshi; Igota, Hiromasa; Matsuura, Yukiko; Azumaya, Munemitsu; Yoshida, Tsuyoshi; Kaji, Koichi; 2019. Effects Of Culling Intensity On Diel And Seasonal Activity Patterns Of Sika Deer (*Cervus nippon*). Scientific Reports 9. DOI: 10.1038/S41598-019-53727-9. | No | None |
| Krauss, Siegfried L.; Roberts, David G.; Phillips, Ryan D.; Edwards, Caroline; 2018. Effectiveness Of Camera Traps For Quantifying Daytime And Nighttime Visitation By Vertebrate Pollinators. Ecology And Evolution 8: 9304-9314. DOI: 10.1002/Ece3.4438. | No | None |
| Carter, Neil; Jasny, Micah; Gurung, Bhim; Liu, Jianguo; 2015. Impacts Of People And Tigers On Leopard Spatiotemporal Activity Patterns In A Global Biodiversity Hotspot. Global Ecology And Conservation 3: 149-162. DOI: 10.1016/J.Gecco.2014.11.013. | No | None |
| Steen, Ronny; Barmoen, Magnus; 2017. Diel Activity Of Foraging Eurasian Red Squirrels (*Sciurus* *vulgaris*) In The Winter Revealed By Camera Traps. Hystrix-Italian Journal Of Mammalogy 28: 43-47. DOI: 10.4404/Hystrix-28.1-11997. | No | None |
| Higdon, Summer D.; Diggins, Corinne A.; Cherry, Michael J.; Ford, W. Mark; 2019. Activity Patterns And Temporal Predator Avoidance Of White-Tailed Deer (*Odocoileus virginianus*) During The Fawning Season. Journal Of Ethology 37: 283-290. DOI: 10.1007/S10164-019-00599-1. | No | None |
| Harmsen, Bart J.; Foster, Rebecca J.; Silver, Scott C.; Ostro, Linde E. T.; Doncaster, C. Patrick; 2009. Spatial And Temporal Interactions Of Sympatric Jaguars (*Panthera onca*) And Pumas (*Puma* *concolor*) In A Neotropical Forest. Journal Of Mammalogy 90: 612-620. DOI: 10.1644/08-Mamm-A-140R.1. | No | None |
| Franco, Marcela; Quijano, Andrea; Soto-Gamboa, Mauricio; 2011. Communal Nesting, Activity Patterns, And Population Characteristics In The Near-Threatened Monito Del Monte, *Dromiciops* *Gliroides*. Journal Of Mammalogy 92: 994-1004. DOI: 10.1644/10-Mamm-A-256.1. | No | None |
| Hicks, NG; Menzel, MA; Laerm, J; 1998. Bias In The Determination Of Temporal Activity Patterns Of Syntopic *Petromyscus* In The Southern Appalachians. Journal Of Mammalogy 79: 1016-1020. DOI: 10.2307/1383110. | No | None |
| Singh, Priya; Macdonald, David W.; 2017. Populations And Activity Patterns Of Clouded Leopards And Marbled Cats In Dampa Tiger Reserve, India. Journal Of Mammalogy 98: 1453-1462. DOI: 10.1093/Jmammal/Gyx104. | No | None |
| Aya-Cuero, Carlos; Rodriguez-Bolanos, Abelardo; Superina, Mariella; 2017. Population Density, Activity Patterns, And Ecological Importance Of Giant Armadillos (*Priodontes maximus*) In Colombia. Journal Of Mammalogy 98: 770-778. DOI: 10.1093/Jmammal/Gyx006. | No | None |
| de las Mercedes Guerisoli, Maria; Caruso, Nicolas; Luengos Vidal, Estela Maris; Lucherini, Mauro; 2019. Habitat Use And Activity Patterns Of *Puma Concolor* In A Human-Dominated Landscape Of Central Argentina. Journal Of Mammalogy 100: 202-211. DOI: 10.1093/Jmammal/Gyz005. | No | None |
| Ghaskadbi, Pallavi; Habib, Bilal; Qureshi, Qamar; 2016. A Whistle In The Woods: An Ethogram And Activity Budget For The Dhole In Central India. Journal Of Mammalogy 97: 1745-1752. DOI: 10.1093/Jmammal/Gyw141. | No | None |
| Swinnen, Kristijn R. R.; Hughes, Nelika K.; Leirs, Herwig; 2015. Beaver (*Castor fiber*) Activity Patterns In A Predator-Free Landscape. What Is Keeping Them In The Dark?. Mammalian Biology 80: 477-483. DOI: 10.1016/J.Mambio.2015.07.006. | No | None |
| Ogurtsov, Sergey S.; Zheltukhin, Anatoliy S.; Kotlov, Ivan P.; 2018. Daily Activity Patterns Of Large And Medium-Sized Mammals Based On Camera Traps Data In The Central Forest Nature Reserve, Valdai Upland, Russia. Nature Conservation Research 3: 68-88. DOI: 10.24189/Ncr.2018.031. | No | None |
| Azlan, JM; Sharma, DSK; 2006. The Diversity And Activity Patterns Of Wild Felids In A Secondary Forest In Peninsular Malaysia. Oryx 40: 36-41. DOI: 10.1017/S0030605306000147. | No | None |
| Cheyne, Susan M.; Macdonald, David W.; 2011. Wild Felid Diversity And Activity Patterns In Sabangau Peat-Swamp Forest, Indonesian Borneo. Oryx 45: 119-124. DOI: 10.1017/S003060531000133X. | No | None |
| Ikeda, Takashi; Uchida, Kenta; Matsuura, Yukiko; Takahashi, Hiroshi; Yoshida, Tsuyoshi; Kaji, Koichi; Koizumi, Itsuro; 2016. Seasonal And Diel Activity Patterns Of Eight Sympatric Mammals In Northern Japan Revealed By An Intensive Camera-Trap Survey. Plos One 11. DOI: 10.1371/Journal.Pone.0163602. | No | None |
| Bu, Hongliang; Wang, Fang; McShea, William J.; Lu, Zhi; Wang, Dajun; Li, Sheng; 2016. Spatial Co-Occurrence And Activity Patterns Of Mesocarnivores In The Temperate Forests Of Southwest China. Plos One 11. DOI: 10.1371/Journal.Pone.0164271. | No | None |
| Bianchi, Rita de Cassia; Olifiers, Natalie; Gompper, Matthew E.; Mourao, Guilherme; 2016. Niche Partitioning Among Mesocarnivores In A Brazilian Wetland. Plos One 11. DOI: 10.1371/Journal.Pone.0162893. | No | None |
| Hearn, Andrew J.; Cushman, Samuel A.; Ross, Joanna; Goossens, Benoit; Hunter, Luke T. B.; Macdonald, David W.; 2018. Spatio-Temporal Ecology Of Sympatric Felids On Borneo. Evidence For Resource Partitioning?. Plos One 13. DOI: 10.1371/Journal.Pone.0200828. | No | None |
| Santos, Fernanda; Carbone, Chris; Wearn, Oliver R.; Rowcliffe, J. Marcus; Espinosa, Santiago; Moreira Lima, Marcela Guimaraes; Ahumada, Jorge A.; Sousa Goncalves, Andre Luis; Trevelin, Leonardo C.; Alvarez-Loayza, Patricia; Spironello, Wilson R.; Jansen, Patrick A.; Juen, Leandro; Peres, Carlos A.; 2019. Prey Availability And Temporal Partitioning Modulate Felid Coexistence In Neotropical Forests. Plos One 14. DOI: 10.1371/Journal.Pone.0213671. | No | None |
| Broeckhoven, Chris; Mouton, Pieter le Fras Nortier; 2015. Some Like It Hot: Camera Traps Unravel The Effects Of Weather Conditions And Predator Presence On The Activity Levels Of Two Lizards. Plos One 10. DOI: 10.1371/Journal.Pone.0137428. | No | None |
| Haidir, Iding Achmad; Macdonald, David Whyte; Linkie, Matthew; 2018. Assessing The Spatiotemporal Interactions Of Mesopredators In Sumatra'S Tropical Rainforest. Plos One 13. DOI: 10.1371/Journal.Pone.0202876. | No | None |
| Hanya, Goro; Otani, Yosuke; Hongo, Shun; Honda, Takeaki; Okamura, Hiroki; Higo, Yuma; 2018. Activity Of Wild Japanese Macaques In Yakushima Revealed By Camera Trapping: Patterns With Respect To Season, Daily Period And Rainfall. Plos One 13. DOI: 10.1371/Journal.Pone.0190631. | No | None |
| Lashley, Marcus A.; Cove, Michael V.; Chitwood, M. Colter; Penido, Gabriel; Gardner, Beth; DePerno, Chris S.; Moorman, Chris E.; 2018. Estimating Wildlife Activity Curves: Comparison Of Methods And Sample Size. Scientific Reports 8. DOI: 10.1038/S41598-018-22638-6. | No | None |
| Doody, J. Sean; McHenry, Colin R.; Rhind, David; Clulow, Simon; 2019. Novel Habitat Causes A Shift To Diurnal Activity In A Nocturnal Species. Scientific Reports 9. DOI: 10.1038/S41598-018-36384-2. | No | None |
| Michalski, Fernanda; Norris, Darren; 2011. Activity Pattern Of *Cuniculus Paca* (Rodentia: Cuniculidae) In Relation To Lunar Illumination And Other Abiotic Variables In The Southern Brazilian Amazon. Zoologia 28: 701-708. DOI: 10.1590/S1984-46702011000600002. | No | None |

TABLE S2 Results from the segmented regressions fitted to accumulation curves of the activity of each study species over the 24-hour cycle. Breakpoint (± 95% confidence interval) refers to the point at which the accumulation curve between activity (i.e., the hour recorded as active) and cumulative number of images reaches an asymptote. R^2^ refers to the fit of the regression. In all cases the number of available images exceeded the number of images at the asymptote), confirming adequate sampling to describe activity.
